# Patient preference in psychological treatment and associations with self-reported outcome: national cross-sectional survey in England and Wales

**DOI:** 10.1186/s12888-015-0702-8

**Published:** 2016-01-15

**Authors:** Ryan Williams, Lorna Farquharson, Lucy Palmer, Paul Bassett, Jeremy Clarke, David M. Clark, Mike J. Crawford

**Affiliations:** Imperial College London, London, UK; Royal Holloway, University of London, London, UK; College Centre for Quality Improvement, Royal College of Psychiatrists, 21 Prescot Street, E1 8BB London, UK; Stats Consultancy, Amersham, UK; University of Oxford, Oxford, UK

**Keywords:** Psychological treatment, Choice behaviour, Patient preference, Psychotherapy, Treatment outcome

## Abstract

**Background:**

Providers of psychological therapies are encouraged to offer patients choice about their treatment, but there is very little information about what preferences people have or the impact that meeting these has on treatment outcomes.

**Method:**

Cross-sectional survey of people receiving psychological treatment from 184 NHS services in England and Wales. 14,587 respondents were asked about treatment preferences and the extent to which these were met by their service. They were also asked to rate the extent to which therapy helped them cope with their difficulties.

**Results:**

Most patients (12,549–86.0 %, 95 % CI: 85.5–86.6) expressed a preference for at least one aspect of their treatment. Of these, 4,600 (36.7 %, 95 % CI: 35.8–37.5) had at least one preference that was not met. While most patients reported that their preference for appointment times, venue and type of treatment were met, only 1,769 (40.5 %) of the 4,253 that had a preference for gender had it met. People who expressed a preference that was not met reported poorer outcomes than those with a preference that was met (Odds Ratios: appointment times = 0.29, venue = 0.32, treatment type = 0.16, therapist gender = 0.32, language in which treatment was delivered = 0.40).

**Conclusions:**

Most patients who took part in this survey had preferences about their treatment. People who reported preferences that were not met were less likely to state that treatment had helped them with their problems. Routinely assessing and meeting patient preferences may improve the outcomes of psychological treatment.

## Background

It has been argued that increasing the amount of choice that patients have can encourage them to take greater interest in their health, increase their adherence to treatment and ensure more cost effective use of available resources [1]. In England efforts to increase patient choice are seen as central to delivering patient-centred care [2], and a number of steps have been taken to give patients greater choice about when and where they are treated [3].

Surveys of patients suggest that many would like greater choice of treatment [4]. Evidence to support claims that providing greater choice to patients increases service quality is limited [5]. While some studies have shown that interventions which support people to make choices about treatment options lead to improved health [6, 7] others have not [8].

Multiple guidelines on the application of evidence-based practise emphasise the use of patient preferences to direct treatment selection, considering it of equal value to symptom profiles, resource availability or past treatment history in guiding management [9, 10] Others speculate that understanding patient preference may improve provider-patient communication, encourage patients to engage with treatment and improve adherence [11, 12].

Much of the literature examining the effect of patient choice on clinical outcomes in mental health focuses on selection between psychological and pharmacological treatment, and yields varied results. Some studies indicate that receiving preferred treatment conveys an additional benefit in terms of clinical measures and treatment retention [13, 14] as well as cost-effectiveness [15], while others find no effect [16, 17]. These contrasting findings may be due to the reluctance of patients with strong preferences to enter controlled trials where they could be randomised to treatment, even those with partial-preference designs [18, 19].

Within the specific context of psychological treatment, patient choice may exert a significant influence due to the large number of variables involved compared with pharmacological interventions. Variations in time, place or therapist, as well as differing modes of psychological therapy, could all affect the therapeutic value of treatment for a specific patient. Meeting patient preferences for some aspects of psychological therapy may lead to lower dropout rates [18, 20]. However, at this stage very little is known about the preferences that people referred for psychological therapy have or the impact that meeting these preferences has on patient-reported outcomes.

The National Audit of Psychological Therapies for Anxiety and Depression was a large scale examination of the practice of psychological therapies in England and Wales [21]. The audit comprised an examination of routine clinical records and a survey of people using a wide range of state-funded primary and secondary care services and compared clinical outcomes and patient experience against agreed standards of care.

The audit was commissioned by the Healthcare Quality Improvement Partnership as part of the National Clinical Audit and Patient Outcomes Programme. The second round of the audit was conducted in 2012–13. At the request of service user representatives on the Project Advisory Group, we added a series of questions on patient preferences to the survey. We analysed data from the audit to determine the prevalence of patient preferences, and the proportion of people who felt that these had been met. We set out to determine the extent to which patients have preferences for psychological treatments and explore what, if any, impact responding to these preferences has on their experiences of treatment.

## Method

All audit data were collected during the second round of the National Audit of Psychological Therapies. A detailed account of methods used in the audit have been published elsewhere [21]. We identified the sample for the audit by contacting medical directors and chief executives of NHS Trusts in England and Head Boards in Wales and asking them to submit contact details for the psychological treatment services they provide. We combined these with contact details from a register of Improving Access to Psychological Therapies services in England, and services that participated in the earlier round of the audit [22]. Prior to the start of the audit we were advised by the National Research Ethics Service and the Ethics and Confidentiality Committee of the National Information Governance Board that formal ethical approval was not required for this quality improvement initiative.

220 services took part in the audit (approximately 60 % of the 350 to 380 services which we estimate were eligible to take part at that time). Each service selected a census date within the period 1^st^ July- 31^st^ October 2012 and all patients receiving treatment from the service on this date were invited to complete an anonymous survey that examined their experience of treatment. Patients were given written information about the audit and had the option of completing either a paper or a web-based version of the questionnaire. Those who opted to complete a paper version were given a pre-paid envelope to return the questionnaire directly to the audit team. In an effort to minimise response bias we made it clear to each participant that the survey was confidential and that the feedback they have could not be traced back to them. We did not seek written informed consent to take part in the survey. Consent was implied when a patient responded to the survey.

Demographic data were not collected from people who did not participate in the survey, but were available from a parallel audit of clinical records that was conducted at the same time.

### Main outcome measure and covariates

The questionnaire for the survey was developed in collaboration with users and providers of psychological treatment services and was piloted prior to the main audit to check that items were understandable and acceptable. For our main outcome measure, respondents were asked to indicate the extent to which they agreed with the statement ‘*this talking treatment helps me cope with my difficulties’ using* a five-point scale (strongly agree, agree, not sure, disagree, strongly disagree).

Patients were also asked five questions about preferences for treatment and whether these preferences had been met. Respondents were asked about preferences about the venue where treatment was delivered, the time of day of appointments, gender of the therapist that they saw, language in which the treatment was delivered (or access to help from an interpreter), and the type of therapy. For each of these features of treatment, respondents were asked to indicate whether it was “not important to me- I have no strong preference”, it was “important to me and I was given enough choice”, it was “important to me but I was not given enough choice” or I am “unsure”. Finally respondents were asked to indicate their age, gender, sexual orientation and ethnicity from a range of categorical options. A full copy of the questionnaire used in the patient survey can be downloaded at:

www.rcpsych.ac.uk/workinpsychiatry/qualityimprovement/nationalclinicalaudits/psychologicaltherapies/psychologicaltherapies/secondroundresources.aspx.

### Statistical methods

We started by calculating the proportion of respondents who had preferences for each of the five items, and whether, among those that had a preference, respondents felt that they were offered enough choice. Data from those who indicated that they were ‘unsure’ were added to those who expressed no preference as we judged that such people were unlikely to have a strong preference.

We then examined univariate associations between whether patients had a preference for the five choice items and demographic characteristics (age, gender, ethnicity and sexual orientation). Finally, we examined associations between whether people felt their treatment had helped them cope with their difficulties (main outcome) and the five choice variables. For this analysis we converted responses on the five-point scale to a dichotomous variable, according to whether patients agreed/strongly agreed that treatment had helped them cope with their difficulties or were not sure/ disagreed/ strongly disagreed.

A feature of the data were that patients were clustered within different services. Outcomes from patients from the same service may be more similar than outcomes from patients from different services. Therefore, to allow for this data structure, all analyses were performed using multilevel statistical methods. Two level models were used with patients nested within services. Due to the binary nature of the outcome, the analysis was performed using multilevel logistic regression. Initially the association between each choice variable and the outcome was examined without considering any possible confounding variables. Subsequently the analysis was repeated, adjusting for demographic variables found to be associated with the choice variables.

## Results

Of 220 psychological therapy services that took part in the audit, 184 (83.6 %) collected data for the patient survey. Patient questionnaires were sent out to 76,950 people and 15,078 (19.6 %) responded. Incomplete returns were removed and data from 14,587(19.0 %) were included in this analysis. Characteristics of those who took part in the study are presented in Table 1 together with aggregate data from the audit of clinical records of people using the 220 treatment services during this period. People who responded to the survey were more likely to be white, female and older than non-responders.

**Table 1:** Demographic characteristics of study participants and comparative data from the case note audit

Demographic characteristics	Study sample n (%)	Sample included in the case notes audit n (%)	Difference in proportions (95 % CI)
Age	*N* = 14148	*N* = 122740	
18 – 24	1088 (7.69)	16405 (13.37)	-5.68 (-5.18, -6.15)
25 - 34	2513 (17.76)	30117 (24.54)	-6.78 (-6.09, -7.44)
35 - 44	3287 (23.23)	28796 (23.46)	-0.23 (-0.51, 0.96)
45 - 54	3519 (24.87)	25359 (20.66)	2.57 (1.84, 3.31)
55 - 64	2474 (17.49)	14269 (11.63)	5.86 (5.22, 6.53)
65 - 74	980 (6.93)	5617 (4.58)	2.35 (1.92, 2.90)
75+	287 (2.03)	2177 (1.77)	0.25 (0.02, 0.51)
Gender	*N* = 13954	*N* = 122585	
Female	9656 (69.24)	79157 (64.57)	4.63 (3.81, 5.43)
Male	4298 (30.76)	43428 (35.43)	-
Ethnicity	*N* = 14004	*N* = 101550	
White	13134 (93.79)	90769 (89.38)	4.41 (3.95, 4.84)
Asian	348 (2.48)	3736 (3.68)	-1.19 (-0.9, -1.47)
Black	159 (1.14)	2788 (2.75)	-1.61 (-1.40, -1.80)
Mixed	219 (1.56)	2181 (2.15)	-0.58 (-0.35, -0.80)
Chinese/Other	144 (1.03)	2078 (2.05)	-1.02 (-0.81, -1.20)

Overall, 86.0 % of patients expressed a preference for at least one aspect of their therapy (*n* = 12549, 95 % CI: 85.5–86.6). 31.5 % of patients expressed at least one preference and felt that they had not been offered sufficient choice (*n* = 4600, 95 % CI: 30.8–32.3). These accounted for 36.7 % of those that expressed at least one preference (95 % CI: 35.8–37.5).

Frequencies and proportion of patients who expressed preferences for each of the five choice variables are presented in Table 2 below. Patients were most likely to have a preference for time of day for their sessions (72.6 %) and least likely to have a preference for accessing therapy in a language other than English or through an interpreter (8.0 %). For each variable, most people who had a preference felt they were offered enough choice, aside from the 4,252 patients who had a preference for the gender of their therapist, of whom 2483 (58.4 %) felt they were not offered enough choice. Demographic factors associated with the likelihood of expressing preferences, taking account of clustering by service are presented in Table 3.

**Table 2:** Proportion of patients expressing preferences for choice of components of psychological treatment

Aspect of treatment	No preference	Expressed a preference	
		Given adequate choice	Not given adequate choice
	n % (95 % CI)	n % (95 % CI)	n % (95 % CI)
Choice of venue	6855 47.7 (46.9-48.5)	5282 36.7 (35.9-37.5)	2242 15.6 (15.0-16.2)
Time of day of appointments	3950 27.4 (26.7-28.1)	8639 59.9 (59.1-60.7)	1837 12.7 (12.2-13.2)
Gender of therapist	10027 70.2 (69.5-71.0)	1769 11.9 (11.4-12.4)	2483 17.9 (16.8-18.0)
Language/ interpreter	11743 92.0 (91.5-92.5)	643 4.9 (4.5-5.3)	382 3.1 (2.8-3.4)
Type of treatment	6844 48.0 (47.2-48.8)	4981 34.9 (34.1-35.7)	2441 17.1 (16.5-17.7)

**Table 3:** Characteristics of patients who expressed a preference for choosing aspects of their psychological treatment (adjusted for clustering by service)

Patient characteristic		Preference for choice of venue	Preference for time of appointments	Preference for gender of therapist	Preference for language/interpreter	Preference for type of therapy
		OR (95 % CI)	OR (95 % CI)	OR (95 % CI)	OR (95 % CI)	OR (95 % CI)
Age	18-24	Reference	Reference	Reference	Reference	Reference
	25-34	1.07 (0.93-1.23)	1.31 (1.10-1.54)*	0.80 (0.69-0.93)*	1.06 (0.79-1.42)	1.16 (1.01-1.34)*
	35-44	1.13 (0.99-1.30)	1.08 (0.92-1.26)	0.76 (0.65-0.88)**	1.12 (0.84-1.49)	1.02 (0.89-1.17)
	45-54	1.11 (0.97-1.27)	0.90 (0.77-1.05)	0.83 (0.72-0.96)*	1.38 (1.05-1.83)*	1.03 (0.89-1.18)
	55-64	1.18 (1.02-1.36)*	0.67 (0.57-0.79)**	0.75 (0.64-0.87)**	1.48 (1.11-1.98)*	0.83 (0.72-0.96)*
	65-74	1.18 (0.99-1.40)	0.47 (0.39-0.57)**	0.74 (0.62-0.90)*	1.76 (1.25-2.47)**	0.67 (0.56-0.80)**
	75+	0.97 (0.75-1.25)	0.40 (0.31-0.53)**	0.54 (0.40-0.73)**	1.71 (1.02-2.86)*	0.41 (0.31-0.54)**
Gender	Male	Reference	Reference	Reference	Reference	Reference
	Female	1.46 (1.36-1.57)**	1.79 (1.65-1.93)**	1.70 (1.56-1.85)**	1.05 (0.91-1.20)	1.37 (1.27-1.47)**
Sexuality	Heterosexual	Reference	Reference	Reference	Reference	Reference
	Gay/Lesbian	0.89 (0.72-1.10)	0.90 (0.72-1.13)	1.42 (1.14-1.76)**	0.61 (0.37-1.00)	*1.31* (1.07-1.63)*
	Bisexual/Other	0.90 (0.72-1.13)	0.90 (0.71-1.15)	1.40 (1.11-1.76)**	1.03 (0.67-1.58)	1.28 (1.02-1.60)*
Ethnicity	White	Reference	Reference	Reference	Reference	Reference
	Asian	1.22 (0.99-1.51)*	1.40 (1.08-1.80)*	1.52 (1.22-1.88)**	3.68 (2.83-4.77)**	1.06 (0.86-1.30)
	Black	1.05 (0.77-1.43)	1.43 (0.98-2.09)	1.39 (1.01-1.92)	1.53 (0.91-2.58)	0.98 (0.72-1.34)
	Mixed	1.16 (0.89-1.51)	1.21 (0.89-1.65)	1.45 (1.10-1.91)*	0.95 (0.55-1.64)	1.29 (0.98-1.68)
	Other	1.24 (0.89-1.72)	1.27 (0.86-1.87)	1.37 (0.97-1.93)	5.44 (3.76-7.85)**	1.69 (1.19-2.38)*

Note: * *p* < 0.05, **p < 0.01 for differences compared to the reference group

Associations between choice variables and patient reported outcomes are presented in Table 4. Patients who reported that they were not given adequate choice were less likely to agree that their treatment helped them cope with their difficulties. Differences between those who had preferences that were met and those with no preferences were less marked aside from for type of therapy and time of day: those reporting that these preferences were met, were more likely to report that therapy had helped them with their problems.

**Table 4:** Proportion of patients who believed treatment had helped them cope with their difficulties according to whether preferences for choice were met

Aspect of treatment	Preference	n / N (%)	Odds Ratio (95 % CI)	*p*-value
Choice of venue	No preference	5769/6776 (85 %)	-	<0.001
	Got preference	4507/5214 (86 %)	1.07 (0.95-1.19)	
	Did not	1491/2218 (67 %)	0.35 (0.31-0.39)	
Time of day of appointments	No preference	3248/3895 (83 %)	-	<0.001
	Got preference	7377/8541 (86 %)	1.22 (1.09-1.36)	
	Did not	1177/1819 (65 %)	0.37 (0.32-0.42)	
Gender of therapist	No preference	8492/9918 (86 %)	-	<0.001
	Got preference	1519/1742 (87 %)	1.09 (0.92-1.28)	
	Did not	1682/2461 (68 %)	0.36 (0.32-0.40)	
Language/ interpreter	No preference	9673/11629 (83 %)	-	<0.001
	Got preference	538/638 (84 %)	1.07 (0.85-1.34)	
	Did not	258/378 (68 %)	0.45 (0.36-0.56)	
Type of treatment	No preference	5762/6768 (85 %)	-	<0.001
	Got preference	4466/4926 (91 %)	1.63 (1.44-1.84)	
	Did not	1452/2416 (60 %)	0.26 (0.23-0.29)	

## Discussion

Data from this survey suggest that three quarters of people who are referred to psychological therapy services for common mental health problems have a preference for when therapy is delivered, and around half have a preference for where and what type of therapy. A significant minority of people have preferences for the gender of the therapist and the language that therapy is delivered in.

The likelihood of patients expressing preferences varies according to demographic factors. As might be expected, patients from certain ethnic groups are more likely to report that accessing therapy in another language or through an interpreter is important to them. Specific ethnic groups (Asian or Mixed) were also more likely to express preferences about the gender of their therapist. Patients who report a sexual orientation other than heterosexual were more likely to express preferences about their therapist’s gender, and about the type of therapy they receive, while women are more likely than men to express preferences about all components of their therapy other than language.

Of those patients who expressed preferences, the majority stated that they were offered adequate choice about this component of their therapy. However, a significant proportion reported that they were not given adequate choice. The exact proportion varied according to the aspect of therapy such that around one in five who had a preference for the time of day felt that this had not been met (17.5 %, *n* = 1837) compared to around a third of those who expressed preferences for venue (29.8 %, *n* = 2242), type of therapy (32.9 %, *n* = 2441) or language (37.3 %, *n* = 382). The preference that was least likely to be met was gender of therapist for which only 1,769 (41.6 %) felt their choice had been met.

The value of providing patients with adequate choice when they express a preference is supported by the findings for patients’ ratings of the extent to which they considered therapy had helped them overcome their problems. Patients who expressed preferences and were not offered adequate choices were less likely to agree that their therapy had helped them, regardless of the component they held preferences for. The size of this effect varies by component- patients who had preferences for type of therapy and were not offered adequate choice were around 6 times less likely to agree that they had been helped than those who were (OR 0.16, 95 % CI 0.14–0.18). Patients who were not offered choices for other components were around 2–3 times less likely, e.g. venue (OR 0.32, 95 % CI 0.29–0.36), time of day (OR 0.29, 95 % CI 0.26–0.32), therapist gender (OR 0.32, 95 % CI 0.27–0.37), language (OR 0.40, 95 % CI 0.30–0.54).

Even more strikingly, for some components (time of day and type of therapy), patients who expressed preferences and received adequate choices were more likely to agree that their therapy had helped than patients with no preferences at all eg. type of therapy (OR 1.69, 95 % CI 1.51–1.91), time of day (OR 1.26, 95 % CI 1.14–1.40).

### Strengths and limitations of the study

Data were obtained from a large, heterogeneous sample of patients from across the whole of England and Wales. Participants were recruited from a variety of services providing differing treatment modalities, incorporating a variety of settings. The outcome measures used were derived from feedback from an expert group of service users and providers. However the study also has several important limitations which must be taken into consideration when interpreting the findings.

Comparative data from a case notes audit conducted in parallel with the survey suggests that the response rate may have been different in different groups of patients [23]. It is possible that people who took time to respond to the survey were more or less likely to have preferences about treatment than those who did not respond. While caution therefore needs to be taken in generalising data on patient preferences to all those using psychological services, the poor response rate is in itself unlikely to affect associations between whether preferences were met and self-reported outcomes.

The study also relied entirely on quantitative data obtained through self-report measures. Other methods, such as qualitative interviews with patients and psychological therapists, may have allowed us to gain more detailed information on the nature of preferences, how they were expressed, reasons why they may not have been met and the possible impact lack of choice may have had on the person’s experience of therapy.

We did not request information about the specific types of therapies that patients preferred when they expected a preference for this aspect of treatment. Three quarters of the respondents were from services funded through the ‘Improving Access to Psychological Therapies’ programme which delivers treatment according to a stepped care model. For these respondents, not being given adequate choice could refer to not being able to choose between low intensity therapy (such as guided self-help) and a traditional high intensity (face-to-face) therapy, or it could refer to not being able to choose between different high intensity therapies.

As the data are cross-sectional we are unable to explore the nature of the association between preferences for treatment and self-reported outcomes. While it is possible that people who had preferences for treatment that were not met went on to experience less benefit from treatment, it is also possible that people who had a poor experience of treatment were more likely to attribute this to their initial preferences not being met when they completed the survey.

Finally, no information is available about diagnoses or other clinical details, and the study was reliant on patient recall of information.

### Implications

Many agencies that have produced guidelines for the treatment of mental illness already stress the importance of understanding patient preferences for treatment options, and where possible using these preferences to guide management decisions [24, 25]. This trend has emerged despite a lack of compelling evidence about whether eliciting and meeting patient preference has an impact on treatment outcomes [26, 27]. Data from this survey suggest that, in relation to the provision of psychological therapies for common mental health problems, efforts to meet patient preferences may have an influence on whether people feel that treatment helps them.

To be successful, psychological therapies require a greater degree of active involvement from patients compared to most other types of pharmacological and medical treatment. They are time consuming, patients must travel regularly to a specific location, and they require patients to form a therapeutic relationship with a therapist [28]. However, no guidance currently exists to indicate how much choice patients should be offered over the conditions and setting of their therapy. Our finding that those who are not offered adequate choices are less likely to agree that their treatment has helped them, highlights the importance of eliciting patient preference in the context of psychological therapy, and where possible offering a choice of options in response.

Our results also imply that particular effort should be made to explore preferences relating to time of day and type of psychological therapy, as offering adequate choice to those with preferences may confer added benefit over those with no preferences. Out of the range of components that we examined, these were also the two where it may be easiest to offer people different options.

In England, the Improving Access to Psychological Therapies (IAPT) initiative has laid out clear guidance for psychological therapy services regarding providing choice of venue, time of appointment, therapist and type of therapy [29] and expects this to be implemented across the UK [30, 31]. At present, the accreditation programme for psychological therapies looks at how these recommendations are being met when services apply for accreditation [32]. The national audit also made recommendations for services to help improve the choice that they offer to patients [21]. At an individual level, prior authors have suggested techniques for addressing patient preferences in psychotherapy [33].

More research is needed to explore the long-term effects of patient choice in psychological therapy. It is difficult to envisage how controlled trials could be devised in which people are randomised to receive or be denied choice of time, place or other aspects of therapy. However prospective observational studies of patients attending different types of services, where more or less choice is available, would be possible and have been recommended as a valuable alternative to randomised controlled trials in these circumstances [34]. These may provide a better guide to the impact that meeting patient preferences have on treatment outcomes. While patient accounts of the impact of therapy are important, such studies should also include standardised outcome measures of mental health.

## Conclusions

This study provides data on the proportion of patients that have preferences for different aspects of the psychological therapy they are offered. The majority of patients would like at least one component of their therapy to be tailored according to their preference. A significant sub-group of these patients feel that they are not offered an adequate range of choice over such elements of their therapy, and that their preferences are not accommodated by their healthcare provider.

We also found that there were demographic differences in the expression of patient preferences and that patients who hold preferences which are not met, are less likely to report that their treatment was helpful. Whilst we are unable to infer a causative relationship between meeting preferences and outcomes, the association between them emphasises the need for further research in this area. We would suggest that any future investigations prospectively examine the impact that failure to both elicit, and respond to, patients’ preferences has on the efficacy of psychological treatments. Such investigations should ideally employ clinical outcome measures, as well as monitoring rates of attendance and attrition.
